# Utilization and Diagnostic Yield of Whole-Body CT in Polytrauma Patients: A Retrospective Cohort Study at a Tertiary Center in Oman

**DOI:** 10.7759/cureus.105230

**Published:** 2026-03-14

**Authors:** Mudhaffar Al-Farras, Aya AalHamad, Mohammed Atta Elmanan, Mamtha Punjee RajaRao

**Affiliations:** 1 Emergency Medicine Department, Sultan Qaboos University Hospital, Muscat, OMN; 2 Emergency Medicine Department, Medical City for Military and Security Services, Muscat, OMN

**Keywords:** life-threatening injuries, polytrauma patient, radiation exposure, trauma team activation, whole-body computed tomography

## Abstract

Background: Whole-body computed tomography (WBCT) is widely used in trauma care, but concerns remain regarding overuse and radiation exposure. This study evaluated WBCT utilization and diagnostic yield among polytrauma patients at Sultan Qaboos University Hospital (SQUH) in Oman. Methods: A retrospective cohort study was conducted on adult polytrauma patients who underwent WBCT between June 2022 and May 2024. The primary outcome was the proportion of negative WBCT scans. Secondary outcomes included predictors of positive WBCT findings. Results: Among 566 patients (444 male (78.4%); mean age, 35.8 years) included in the study, 245 patients (43.3%) had negative WBCT scans. Positive scans were associated with hypotension (systolic blood pressure (SBP) <90 mmHg; OR 3.88), low Glasgow Coma Score (GCS) (<13; OR 0.16), hypoxia (oxygen saturation (SpO₂) <94%; OR 6.95), and high injury severity score (ISS) (>24; OR 12.14). Trauma activation cases had a higher positivity rate (74.5%, 223 patients) compared to non-activation cases (36.2%, 74 patients). Conclusion: WBCT remains valuable for detecting multisystem injuries, but selective use based on physiological markers and mechanism of injury is recommended to reduce unnecessary radiation exposure.

## Introduction

Trauma remains a leading cause of morbidity and mortality worldwide, particularly among the younger population. According to the World Health Organization, injuries account for approximately 10% of global deaths annually, with road traffic accidents being the predominant cause [1].

In the critical early phase of trauma care, often referred to as the 'golden hour', rapid and accurate diagnosis is essential to guide timely interventions and improve patient outcomes. Whole-body computed tomography (WBCT), also known as pan-scan or total-body CT, has emerged as a pivotal diagnostic tool in the management of patients with major trauma. Unlike the traditional selective CT (SCT) approach, which relies on clinical judgment and focused imaging, WBCT enables a comprehensive assessment of the head, neck, chest, abdomen, pelvis, and spine in a single session. This facilitates the early detection of life-threatening injuries, including those that may be clinically occult [2,3].

Numerous studies have demonstrated the clinical benefits of WBCT. Meta-analyses and large cohort studies have shown that WBCT is associated with reduced time to diagnosis, shorter emergency department stays, and improved survival rates, particularly in patients with severe or multiple injuries [4-6]. Notably, Huber-Wagner et al. reported that WBCT significantly reduced mortality in haemodynamically unstable patients, with a number needed to scan (NNS) of 20 to save one life in patients with severe shock [7]. Despite these advantages, concerns persist regarding the increased radiation exposure associated with WBCT and its potential long-term risks, such as radiation-induced malignancies [1,7]. However, advancements in CT technology, including dose-reduction protocols and iterative reconstruction techniques, have significantly mitigated these risks [8].

International guidelines increasingly support the use of WBCT in trauma settings. Countries such as Australia, Malaysia, Sweden, and the United Kingdom have developed protocols recommending WBCT for patients with high-energy mechanisms of injury or compromised physiological parameters [3,9,10]. Nevertheless, the debate continues regarding the optimal patient selection criteria for WBCT. While some advocate for its routine use in all major trauma cases, others recommend a more selective approach to balance diagnostic yield against potential harm [11-13]. This underscores the need for further research to refine clinical decision-making tools and establish evidence-based thresholds for WBCT utilization. Therefore, for the purpose of this study, polytrauma patients are defined as patients who sustain injuries to multiple body regions or are involved in a high-risk mechanism of injury that requires trauma team evaluation and WBCT. The primary endpoint highlights the proportion of negative WBCT in polytrauma patients, which reflects the diagnostic yield of whole-body imaging. Meanwhile, the secondary endpoint evaluates the relationship between the initial clinical parameters and positive WBCT findings, which will assess the factors that influence the appropriateness of WBCT utilization and support improved patient selection. In summary, this study aims to evaluate both the diagnostic performance and real-world utilization of WBCT in trauma care.

## Materials and methods

Study design and setting

This was a retrospective cohort study, including all adult polytrauma patients who presented to the Emergency Department (ED) of Sultan Qaboos University Hospital (SQUH) and underwent WBCT as part of their initial trauma assessment. SQUH is a tertiary academic center located in Muscat, Oman, with an annual ED census of approximately 60,000 visits. The study was approved by the Medical Research Ethics Committee (MREC), College of Medicine and Health Sciences, Sultan Qaboos University (reference: SQU-EC/ 156\2024 MREC # 3334), with a waiver of informed consent granted due to the retrospective nature of the study.

Trauma patients presenting to the ED were triaged according to clinical severity. Patients without features suggestive of significant trauma were managed by emergency physicians, whereas those classified as high-acuity cases or brought in by paramedics with suspected major trauma were managed under the hospital’s trauma activation protocol. Trauma team activation was based on predefined institutional criteria and clinical judgment.

Study population

**Figure 1:**
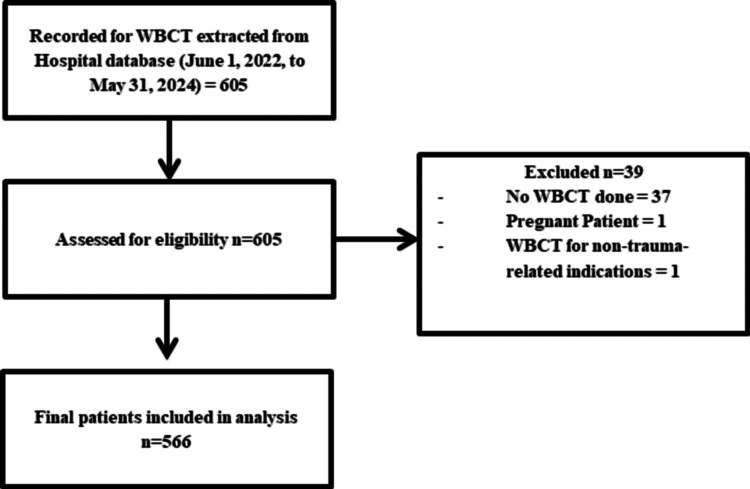
Recruitment flowchart of patients involved in the study WNCT: whole-body computed tomography

The study included all adult patients aged 18 years or older who underwent WBCT during their initial evaluation for polytrauma between June 1, 2022, and May 31, 2024. Polytrauma patients are defined as patients who were exposed to injuries to multiple body regions or involved in a high-risk mechanism of injury with physiological abnormalities that prompt trauma team activation and WBCT imaging. Patients were excluded if they were younger than 18 years, pregnant, deceased prior to undergoing WBCT, or if WBCT was performed for indications unrelated to trauma. After applying these criteria, a total of 566 patients were included in the final analysis (Figure 1).

Initial trauma management

Upon arrival at the ED or following prehospital handover, patients were assessed by the emergency medicine team leader to determine the need for trauma team activation. This decision was guided by institutional trauma protocols and took into account the mechanism of injury, hemodynamic status, and findings from the primary survey. Patients who were initially stable or deemed non-urgent were managed by the ED team, while those with suspected major trauma underwent coordinated multidisciplinary evaluation.

Although a formal validated WBCT decision rule was not implemented during this study period, the imaging followed the institutional trauma activation practice, which involves the clinical presentation of those patients. Such presentations included: high-risk mechanisms of injury such as high-speed or rollover motor vehicle collisions, ejection from a vehicle, falls from heights exceeding six meters, or cases involving severe injury or death of another occupant. Additional clinical considerations prompting WBCT included suspected injuries involving multiple body regions, abnormal vital signs, reduced level of consciousness, endotracheal intubation or procedural sedation, evidence of intoxication, or an unreliable physical examination due to agitation or altered mental status.

Trauma team leadership was initially assumed by the emergency medicine attending physician and subsequently transitioned to the trauma surgery consultant. The trauma team consisted of personnel from trauma surgery, anesthesiology, and radiology, with additional specialty consultations, including orthopedic surgery, neurosurgery, vascular surgery, thoracic surgery, and plastic surgery, obtained as clinically indicated.

Imaging protocol and image interpretation

The institutional WBCT protocol consisted of a non-contrast CT of the head and neck, followed by contrast-enhanced arterial phase imaging of the chest, abdomen, and pelvis, and a venous phase of the abdomen and pelvis. Imaging coverage extended from the vertex of the skull to the ischial tuberosities, excluding the upper and lower extremities. Intravenous contrast was administered at a dose of 1.5-2.0 cc/kg using an automated injector through peripheral venous access. After completion of the primary survey and patient stabilization, the WBCT imaging was typically performed whenever the patients were deemed stable to be transferred to the CT room. All WBCT scans were initially interpreted by the on-call radiologist and subsequently reviewed and verified by a radiology attending, as part of routine departmental quality assurance practice. Meanwhile, in case of discrepancy, the attending radiologist’s report served as the final interpretation. Imaging findings were categorized according to anatomical regions, including the head, spine, chest, abdomen, and pelvis. A WBCT was classified as positive if any acute traumatic injury was identified in one or more regions, irrespective of the injury severity or need for intervention. Conversely, a WBCT was considered negative if no acute traumatic injuries were detected across all evaluated regions from head to pelvis.

Outcomes and statistical analysis

The primary outcome of the study was the proportion of polytrauma patients with negative WBCT findings. The secondary outcome was the association between initial clinical parameters and positive WBCT results.

Statistical analysis was performed using the IBM SPSS Statistics for Windows, version 30.0.0 (IBM Corp., Armonk, New York, United States). Categorical variables were summarized as frequencies and percentages, while continuous variables were reported as means with standard deviations (SDs) for normally distributed data. Comparisons between categorical variables were conducted using the Chi-square test. Univariate logistic regression analysis was used to identify clinical predictors of negative WBCT findings across different anatomical regions, with results presented as odds ratios (ORs) and corresponding 95% confidence intervals (CIs). A p-value of ≤0.05 was considered statistically significant. Missing data were assessed prior to analysis, and the variables with incomplete documentation were analyzed using available case analyses. The proportion of missing observations was reported whenever it was relevant. There was no data imputation due to the retrospective nature of the dataset.

## Results

Descriptive analysis

A total of 566 patients were included in our study. Of these, 444 (78.4%) were male, and 122 (21.6%) were female. The mean age of patients was 35.8 ±12.4 years, with no significant difference between male (35.9 ± 12.4) and female (35.5 ± 12.3) patients. The majority sustained blunt trauma (n=542, 95.6%), most commonly from motor vehicle collisions (MVC) (n=352, 64.9%), followed by pedestrian injuries (n=101, 18.6%) and falls (n=89, 15.9%). Penetrating injuries accounted for 24 cases (4.4%), predominantly due to assault (n=17, 70.8%) and stabbing (n=7, 29.2%). Nearly half of the patients (n=297, 52.5%) required trauma team activation, while the remainder (n=269, 47.5%) were initially assessed by emergency physicians. Intoxication was documented in 7.4% of patients (n=42). The mean of the initial systolic blood pressure (SBP) was 132.3 ± 22.0 mmHg, and the initial mean pulse rate was 94.9 ± 18.0 beats per minute. The mean respiratory rate was 20 ± 5.3 breaths per minute, and the oxygen saturation (SpO_2_) was 96% ± 6.4. The mean Glasgow Coma Score (GCS) was 13.8 ± 2.6; which was sub-classified into: mild (13-15 score range) with 495 patients (87.5%), moderate (9-12 score range) with 21 patients (3.7%), and severe (3-8 score range) with 50 patients (8.8%) were in the severe category (Table 1 and Table 2).

**Table 1:** Data (continuous variables) of the trauma patients included in the study (N=566)

Variable	Mean ± SD
Age (years)	35.8 ±12.4 years
Systolic Blood Pressure (mmHg)	132.30 ± 22.02
Respiratory Rate (breaths per minute)	20.31 ± 5.34
SpO2	96.99 ± 6.43

**Table 2:** Data (categorical variables) of the trauma patients included in the study (N=566) E-FAST: extended focused assessment with sonography for trauma; GCS: Glasgow Coma Score; ISS: injury severity score

Variable	Frequency (Percentage)
Sex	
Male	444 (78.4 %)
Female	122 (21.6 %)
Mechanism of Injury	
Blunt	542 (95.6%)
Penetrating	24 (4.4 %)
E-FAST	
Positive	28 (5%)
Negative	245 (43.2%)
Not Documented	293 (51.8%)
GCS Categories	
Mild category (13–15 score range)	495 (87.5%)
Moderate category (9–12 score range)	21 (3.7%)
Severe category (3–8 score range)	50 (8.8%)
ISS Categories	
1–8	302 (53.4%)
9–15	127 (22.4%)
16–24	56 (9.9%)
>24	81 (14.3%)
Nationality	
Omani	320 (56.5 %)
Non-Omani	246 (43.5 %)
Intoxication on presentation	
Yes	42 (7.4 %)
No	524 (92.6 %)
Trauma activation level	
Activated trauma team	297 (52.5 %)
Assessed primarily by ED physician (no activation)	269 (47.5 %)

ED disposition showed that 65 patients (11.5%) were admitted to the intensive care unit (ICU), 65 patients (11.5%) were immediately taken to the operating theatre, 232 patients (41%) were admitted to the ward, and 204 patients (36%) were discharged after being observed and cleared in the ED (Table 3).

**Table 3:** Disposition of trauma patients on presentation to the emergency department (ED)

ED Disposition	Number of Patients	Percentage
Admitted to Intensive Care Unit	65	11.5
Taken directly to Operating Theatre	65	11.5
Admitted to Ward	232	41.0
Discharged after observation and clearance in ED	204	36.0
Total	566	100.0

The median injury severity score (ISS) was classified into four categories, each category representing a scale. A total of 302 patients (53.4%) had ISS scores in the range of 1-8, 127 patients (22.4%) had ISS scores in the range of 9-15, 56 patients (9.9%) had ISS scores in the range of 16-24, and 81 patients (14.3%) had ISS scores greater than 24. Extended focused assessment with sonography in trauma (E-FAST) was documented in 273 patients (48.2%), with 28 patients (4.9%) yielding positive findings. The mean arterial pH was 7.35 ± 0.07 among 380 (67%) trauma patients, and elevated lactate (> 2) in 369 patients (65.2%).

Outcome analysis

**Figure 2:**
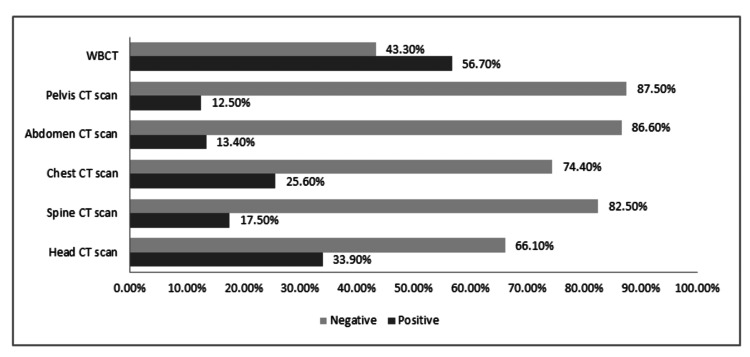
Distribution of negative versus positive scans per region WBCT: whole-body computed tomography

**Figure 3:**
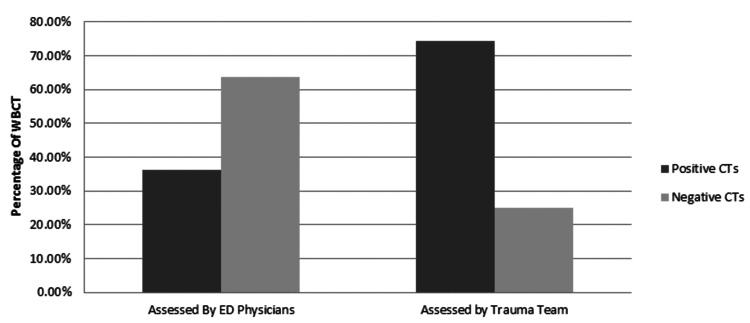
Comparison of negative scans vs. positive scans in patients assessed by ED physicians vs trauma team WBCT: whole-body computed tomography

**Figure 4:**
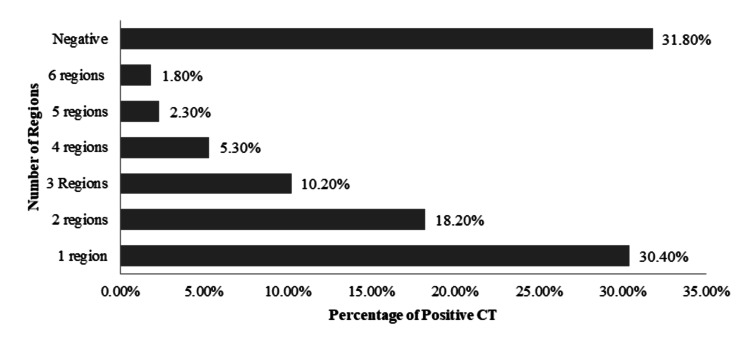
Sub-classification of positive scans based on the number of anatomical body regions affected radiologically

Negative WBCT scans were seen 43.3% (n=245) of patients, while the positive rate was seen in 56.7% (n=321). Figure 2 highlights the rate of positive versus negative scans per region. Further analysis revealed that the rate of negative CTs compared to positive CTs in patients assessed by emergency physicians was 63.80% (n=172) vs. 36.20% (n=97), while the rate of negative CTs versus positive CTs in patients assessed by the trauma team was 24.90% vs. 74.50% (Figure 3). The positive scans were further sub-classified based on the number of anatomical body regions that were affected radiologically. One body region was affected in 172 patients (30.4%), two body regions were affected in 103 patients (18.2%), and three or more body regions were affected in 111 patients (19.6%). Musculoskeletal structures were positive in 214 patients (37.8%) (Figure 4).

As part of the secondary analysis, correlations between initial clinical parameters and WBCT outcomes were examined. Low SBP was significantly associated with positive WBCT findings, with affected patients demonstrating nearly a four-fold increase in the odds of detecting traumatic injuries on CT (OR 3.88, 95%CI 1.46-10.32; p<0.01). A reduced GCS score also showed a strong association with CT positivity, supported by a significant chi-square test (χ²=21.9, p<0.001) and an inverse relationship reflected by an OR of 0.16 (95%CI 0.07-0.38). Similarly, oxygen saturation below 94% on presentation was a strong predictor of positive WBCT findings, with nearly seven-fold higher odds of detecting traumatic injuries (χ²=28.5, p<0.001; OR 6.95, 95%CI 3.10-15.58). Higher ISS values demonstrated the strongest association with CT positivity, showing a highly significant correlation (χ²=49.6, p<0.001) and more than a 12-fold increase in the odds of positive WBCT findings (OR 12.14, 95%CI 5.19-28.42). In contrast, intoxication status was not significantly associated with WBCT outcomes and did not demonstrate a meaningful correlation with CT positivity (p=0.48).

## Discussion

This retrospective cohort study explored clinical characteristics, imaging outcomes, and predictors of positive WBCT findings among trauma patients presenting to the ED in a tertiary hospital in Oman. The predominance of young male patients reflects regional and global trauma epidemiology, as demonstrated in Table 1, where high-energy blunt mechanisms, particularly road traffic collisions, remain the primary contributors to serious trauma [2,12].

The diagnostic yield in this study (56.7%) aligns with rates reported internationally, where WBCT positivity typically ranges between 30% and 70% [1,2,4,5]. The finding that almost 20% of positive scans involved injuries across three or more regions reinforces the established role of WBCT in identifying clinically occult multisystem trauma [2,4,6]. Furthermore, our results showed that there is predominance of head, spine, and chest injuries, as highlighted in Figure 2, which mirrors the classical blunt trauma distribution described in multicenter WBCT studies [6,7]. Although extremity CT is not routinely included in WBCT protocol, the high proportion of musculoskeletal injuries (37.8%) in this cohort is consistent with previous reports and supports the use of targeted limb imaging when indicated [11,12].

**Figure 5:**
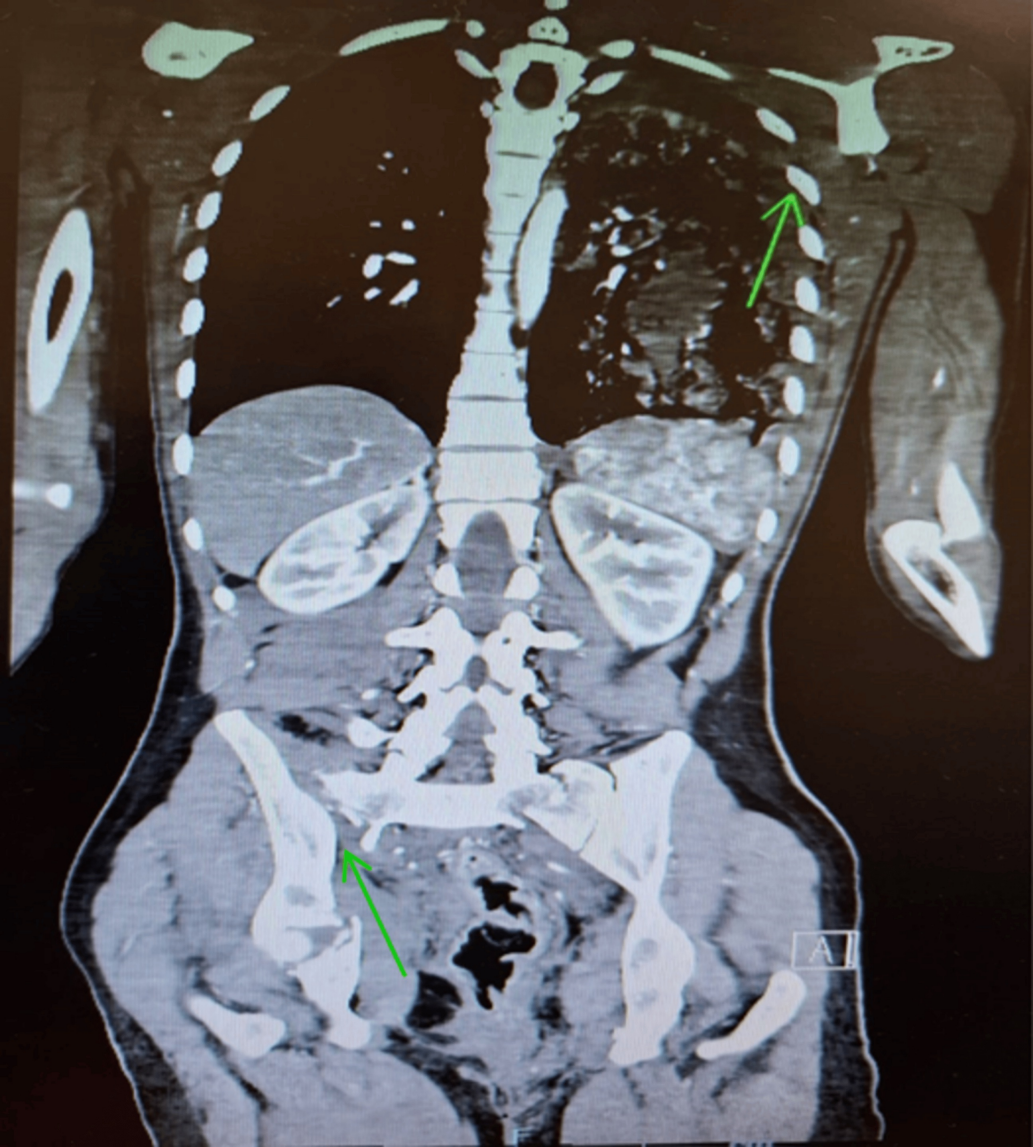
Contrast-enhanced CT demonstrating left hemo-pneumothorax, bilateral pulmonary contusions with intraparenchymal hemorrhage, left rib fractures, and complex pelvic and sacral fractures.

**Figure 6:**
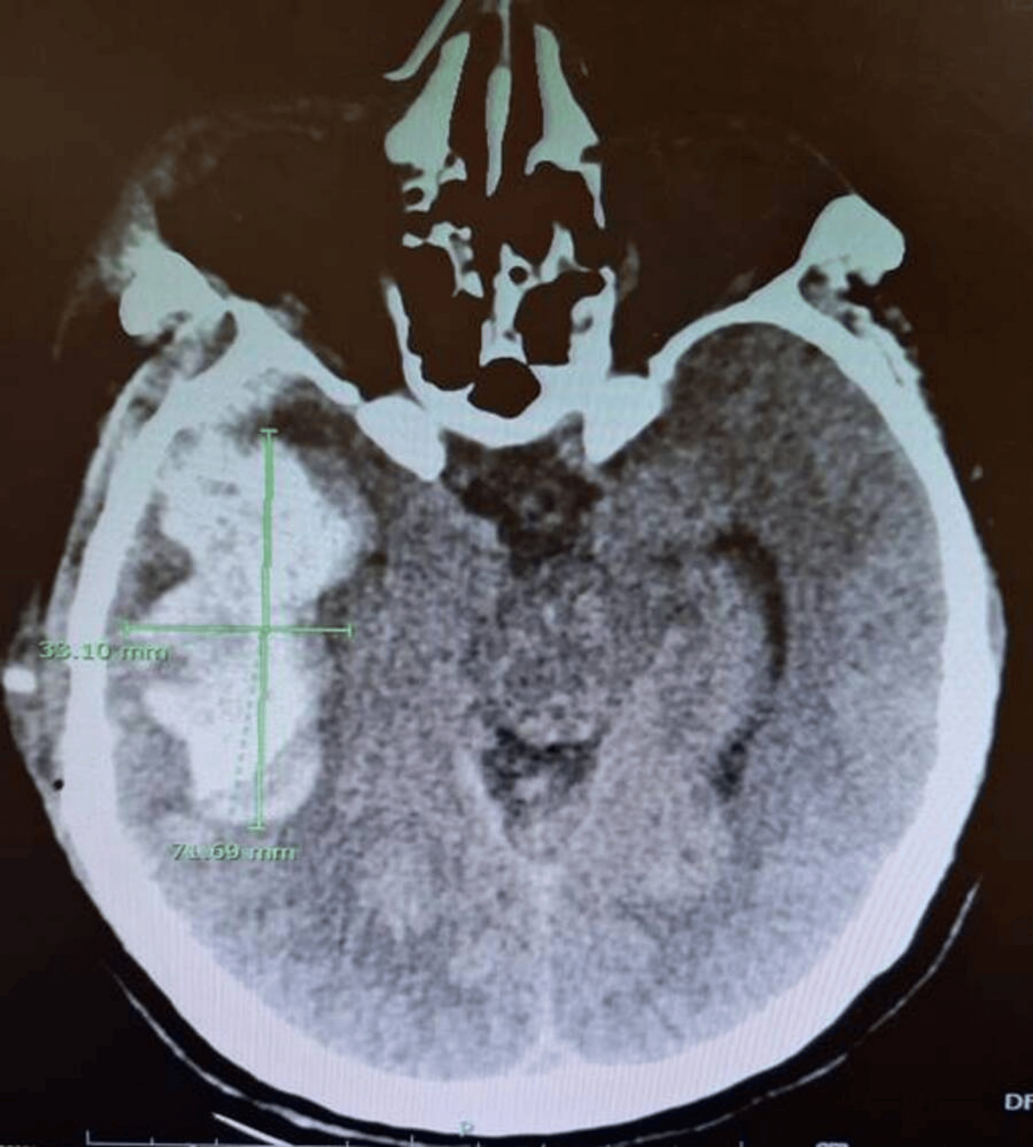
CT brain demonstrating large right parietotemporal hematoma.

Several physiological markers correlated strongly with positive imaging, where hypotension (SBP <90 mmHg) was associated with higher odds of detecting the likelihood of significant intra-torso injury; in our study, it was associated with nearly a four-fold higher odds of CT positivity. This is consistent with existing trauma literature establishing SBP as a surrogate for major hemorrhage [6,7]. Figure 5 demonstrates a CT image of a trauma patient with multiple injuries in various regions and presenting with hypotension. Low GCS strongly predicted (p<0.001; OR 0.16, 95%CI 0.07-0.38) intracranial and multi-system injury, in line with prior WBCT outcome analyses [5,6]. Figure 6 illustrates a CT image of the head region with positive findings of traumatic brain injury in a trauma patient with low GCS.

Furthermore, hypoxia (SpO₂ <94%) emerged as having a strong association with positive WBCT in this cohort (p<0.001; OR 6.95, 95% CI 3.10-15.58), supporting previous work indicating that respiratory compromise is an early marker of thoracic or multisystem trauma [3,9]. Elevated lactate and low arterial pH, established markers of global hypoperfusion, similarly showed strong predictive value, echoing findings from multiple WBCT decision frameworks [1,10]. Higher ISS categories naturally correlated with higher WBCT positivity (p<0.001; OR 12.14, 95%CI 5.19-28.42), as demonstrated across major WBCT survival and diagnostic studies [2,4,5]. Collectively, these findings suggest that physiological instability may be useful when considered alongside clinical judgment in imaging decisions.

The markedly higher positivity rate among trauma activation cases (74.5%), as illustrated in Figure 3, is consistent with international data showing improved diagnostic precision when WBCT is selectively used among high-acuity patients [3,9,10]. Even in the absence of a formalized institutional WBCT protocol, this validates the existing activation criteria. However, a 43.3% negative WBCT rate highlights the well-documented challenge of balancing over-triage against early detection of life-threatening injuries [2,12]. This mirrors international concerns about radiation exposure, cost, and overuse, particularly in hemodynamically normal patients [8,11].

The inconsistent use of E-FAST in this cohort warrants attention. E-FAST was documented in fewer than half (48.2%) of the patients, and its positivity rate was notably low at 4.9%. This limited deployment contrasts with current guidelines, which underscore E-FAST as an essential adjunct, particularly for unstable patients or in settings where access to CT is delayed or restricted [14]. The under-utilization observed may reduce the effectiveness of early trauma assessment, potentially impacting the timely identification of life-threatening thoracic and abdominal injuries. Improved integration of E-FAST into trauma protocols could enhance early clinical decision-making. By ensuring consistent documentation and use, emergency physicians and surgeons may better leverage E-FAST’s rapid bedside diagnostic capabilities to complement other imaging modalities, ultimately supporting more efficient and targeted patient care in acute trauma scenarios.

Interestingly, intoxication was not associated with higher positivity rates (p=0.48), supporting the growing argument that intoxication alone should not drive liberal WBCT use, a finding echoed in recent studies advocating more evidence-based approaches [1,2,11]. The convergence of hemodynamic abnormalities, impaired consciousness, metabolic derangements, and high-energy mechanisms as predictors supports the need for a structured WBCT decision framework. At the same time, the substantial proportion of negative scans underscores the risk of unnecessary radiation and resource overuse, long-standing concerns emphasized in prior international guidelines and meta-analyses [1,8,10,13].

These results collectively indicate an opportunity to design local imaging protocols to improve diagnostic efficiency while minimizing avoidable exposure. Several recommendations can be implemented to improve future approaches, and this includes: (i) Validated predictors, including GCS <13, SBP <90 mmHg, SpO₂ <94%, elevated lactate, low pH, and high-energy mechanism, could be included in a standardized WBCT decision pathway. Similar structured tools have been endorsed in international WBCT guidelines and meta-analyses to reduce unnecessary scanning and ensure high-risk patients are appropriately imaged [1,3,9,10,13]. (ii) A selective regional CT approach can be adopted for physiologically stable, low-risk patients, reserving WBCT for those with instability or multiple red flags. This aligns with recommendations from European and Australasian trauma systems advocating risk-stratified imaging to balance diagnostic accuracy with radiation stewardship [3,9,11]. (iii) Given the high positivity rate among activation cases, periodic auditing and refining of trauma activation criteria can further optimize patient selection, consistent with international trauma guidelines [3,9,10]. (iv) Because intoxication alone was not predictive of injury, imaging decisions should be anchored in physiological and mechanistic factors, reflecting recent evidence discouraging routine WBCT use solely on the basis of altered mental status [11]. (v) Protocolized and systematic E-FAST use should be reinforced. International guidelines emphasize E-FAST as a rapid, low-risk modality essential for early identification of thoracic and abdominal injuries, especially when CT access is delayed [1,3,9]. (vi) Regular audits of WBCT utilization, diagnostic yield, radiation burden, and clinical impact will enable iterative improvement and ensure alignment with global best practices in trauma imaging [8,10]. (vii) Ongoing collaborative training among emergency physicians, trauma surgeons, radiologists, and pre-hospital teams is essential for optimizing recognition of high-risk patients and ensuring consistent application of evidence-based imaging strategies [3,9].

In summary, this study has notable strengths, including the large cohort of trauma patients who have been managed in a tertiary academic center, reflecting real-world trauma practice. Additionally, it highlights clinically meaningful outcomes and identifies the physiological findings that are strongly related to the WBCT positivity, such as SBP, oxygen saturation, ISS, and GCS, where all such findings are consistent with international trauma literature.

Study limitation

This study has several limitations. First, this was a retrospective study conducted in a single center, which reflects the institutional practice pattern, which may limit generalizability to other trauma centers. Furthermore, WBCT utilization was guided by clinical judgment rather than a standardized decision rule, which may introduce selection bias reflective of real-world practice. In addition, only univariate logistic regression analysis was performed; therefore, potential confounding between clinical variables could not be fully adjusted. Multivariable modeling may better identify independent predictors of WBCT positivity in future prospective investigations. Furthermore, WBCT positivity was defined as the presence of any acute traumatic finding without formal injury severity grading, which may influence interpretability and generalizability across different trauma systems. Furthermore, the cost-effectiveness and radiation exposure metrics weren’t highlighted, which might be beneficial in assessing the appropriateness of WBCT utilization. Finally, patient-centered outcomes such as mortality, missed injuries, and long-term outcomes were not addressed in this study, but can be explored in future prospective investigations.

## Conclusions

This study provides knowledge about the real-world utilization of the WBCT in trauma evaluation within a tertiary care centre. Despite showing its diagnostic yield, the high proportions of negative WBCT highlight existing opportunities that will further refine patient selection. Nevertheless, physiological indicators such as hypotension, reduced GCS, hypoxemia, and higher ISS showed significant correlation with WBCT positivity, which suggests that those parameters can be utilized practically during clinical assessment when considering the need for comprehensive imaging. Ultimately, incorporating clinical assessment, physiological indicators, mechanism of injury, and bedside assessment tools (such as E-FAST) into structured imaging pathways may enhance diagnostic efficiency while reducing unnecessary radiation exposure and resource utilization.
